# An Evaluation of US Food and Drug Administration’s Program to Register HIV Drugs for Use in Resource-Constrained Settings

**DOI:** 10.1001/jamanetworkopen.2019.15787

**Published:** 2019-11-20

**Authors:** Harinder Singh Chahal, Kalli Koukounas, Peter Capella, Ryan Presto, Jeffrey S. Murray, Martin Shimer, Karen Riley, Mary Lou Valdez

**Affiliations:** 1 Office of Public Health Strategy and Analysis, Office of the Commissioner, Food and Drug Administration, Silver Spring, Maryland; 2 Now with McKinsey & Company, New York, New York; 3 Office of Pharmaceutical Quality, Center for Drug Evaluation and Research, Food and Drug Administration, Silver Spring, Maryland; 4 Office of Generic Drugs, Center for Drug Evaluation and Research, Food and Drug Administration, Silver Spring, Maryland; 5 Office of New Drugs, Center for Drug Evaluation and Research, Food and Drug Administration, Silver Spring, Maryland; 6 Office of Global Policy and Strategy, Office of the Commissioner, Food and Drug Administration, Silver Spring, Maryland

## Abstract

**Importance:**

The US Food and Drug Administration (FDA) program to review antiretroviral drugs for use in low-resource settings via the US President’s Emergency Plan for AIDS Relief (PEPFAR) now supports treatment of more than 14 million patients with HIV. However, an in-depth evaluation of the program has not been undertaken.

**Objective:**

To conduct a quantitative analysis of the FDA-reviewed antiretroviral drug applications in order to assess the contributions of PEPFAR and to identify areas for improvement.

**Design, Setting, and Participants:**

A cross-sectional study was conducted of all PEPFAR applications submitted to the FDA from December 1, 2004, to May 31, 2018. The analyses were conducted between October 2018 and February 2019.

**Main Outcomes and Measures:**

Numbers and types of applications reviewed, how long it took for applications to obtain approval or tentative approval (time to registration), how often the FDA issued a complete response letter (CRL) identifying deficiencies precluding application approval or tentative approval and their reasons, and the association between CRLs and time to registration.

**Results:**

Overall, 260 PEPFAR applications for 327 antiretroviral therapies were reviewed by FDA, of which 216 applications (83%) for 272 drugs were authorized for use. Of the 216 authorized applications, 184 applications for 231 drugs remain in active status and, thus, are available for use. Twenty-six percent (56 of 216) of the applications were for pediatric-specific formulations or strengths; the remainder were for adults. For all 216 applications, the median (interquartile range) time to registration was 10.0 (7.0-17.5) months. Thirty-seven percent (95 of 260) of the applications received 1 or more CRLs, resulting in a total of 172 CRLs; most applications received 1 CRL, whereas some were issued up to 6 CRLs. Among all CRLs, 264 deficiency reasons were identified; the most common deficiencies were associated with manufacturing processes (155 [44%]), followed by product labeling (62 [23%]), and failing facility inspections (54 [20%]). Complete response letters were associated with an increased time to registration. Applications without CRLs had a median (interquartile range) time to registration of 9.0 (5.5-12.0) months, whereas those with at least 1 CRL took a median (interquartile range) of 22.0 (14.0-38.0) months (*P* < .001).

**Conclusions and Relevance:**

The FDA’s PEPFAR program has made many antiretroviral drugs available for global use. However, FDA and the pharmaceutical companies could take steps to improve the quality of applications submitted to prevent avoidable deficiencies in manufacturing processes and labeling. Further efforts to develop better, easier to use pediatric-specific therapies are needed.

## Key Points

**Question:**

What has the US Food and Drug Administration (FDA) program to review antiretroviral drugs for use in low-resource settings via the US President’s Emergency Plan for AIDS Relief (PEPFAR) achieved since its inception in 2004, and how could the program be more effective?

**Findings:**

This cross-sectional study found that under PEPFAR, the FDA has authorized 216 applications for 272 antiretroviral drugs, including 26% for pediatric use. Ninety-five of 260 applications (37%) received 172 rejection letters, primarily for deficiencies in manufacturing processes (44%).

**Meaning:**

The FDA’s efforts have made many HIV drugs available for use globally; however, more pediatric-specific therapies are needed, and the quality of applications needs to improve.

## Teaser

This cross-sectional study analyzes the contributions of the US Food and Drug Administration (FDA) program for reviewing antiretroviral drugs for use in low-resource settings, as well as areas for improvement.

## Introduction

The HIV/AIDS epidemic is global; in 2018, approximately 38 million patients were living with HIV/AIDS, 1.7 million individuals became newly infected with HIV, and 770 000 infected individuals died.^1^ The United States established the President’s Emergency Plan for AIDS Relief (PEPFAR) in 2003 to fight HIV globally through a combination of treatment, care, and prevention programs.^2^ Since its inception, PEPFAR has helped provide broad access to antiretroviral therapy (ART) in more than 50 resource-constrained countries.^3^ Of the 21.7 million patients with access to treatment in 2018, more than 14 million, or 38% of all persons living with HIV, were supported with PEPFAR ARTs.^1,3^

In May 2004, the US Food and Drug Administration (FDA) announced a new initiative to help ensure that PEPFAR’s ARTs are safe, effective, and subject to rigorous quality review.^3^ Through guidance and outreach to industry, FDA encouraged all manufacturers to submit US marketing applications for single-molecule drugs, fixed-dose combinations, and copackaged versions of previously approved ARTs, including new formulations, even for those medications with patents or other market protections in place in the United States.^4,5,6^

The US Department of State, which oversees PEPFAR, decided that ARTs used by the program should meet the same standards as drugs sold in the United States and, thus, should be reviewed by FDA before being available for PEPFAR procurement.^7^ Applicants are required to submit Abbreviated New Drug Applications (ANDAs) for medicines that are duplicates of existing drug products (single or multidrug combinations) that have already been approved for marketing in the United States, except for differences permitted by FDA regulations.^4^ This is the same path to market for generic drugs sold in the United States.^4^ Similarly, if an applicant is proposing a new variation of previously approved ARTs, such as a new fixed-dose combination of 2 or more existing products, new strengths, or new formulations of these drugs that might make HIV therapy simpler or easier to use, they must submit a New Drug Application (NDA), as they would when seeking US marketing authority.^4^

Products that still have US marketing protections because of patents or other intellectual property rights receive a tentative approval from the FDA rather than a full approval.^4^ Tentative approval signifies that the product meets all safety, efficacy, and manufacturing quality standards for marketing in the United States but cannot be sold in the United States because of legal market protections.^4^ Once the ARTs are approved or tentatively approved, they become available for procurement by PEPFAR and other entities.

Other global entities, such as the World Health Organization (WHO) and the Global Fund to Fight AIDS, Tuberculosis, and Malaria (Global Fund), also use FDA-reviewed ARTs to enhance their respective lists of quality-assured ARTs, thus amplifying the downstream outcomes of the FDA program.^8^ In addition, FDA-reviewed ARTs can also be used to support HIV treatment guidelines developed by WHO and the HIV community, which includes recommendations on first-line or second-line ARTs, depending on patient needs.^8,9^

Previous literature^4,8,10^ has analyzed the outcomes of FDA PEPFAR ARTs on treatment costs and the downstream effects of FDA-reviewed drugs via WHO and the Global Fund. This study provides an in-depth quantitative analysis of the ART applications and products reviewed by FDA to better understand the agency’s PEPFAR contributions and to identify potential areas for improvement.

## Methods

We conducted a cross-sectional study to characterize all applications and products submitted to FDA under the PEPFAR program from December 1, 2004 (beginning of the program), to May 31, 2018. For simplicity, in this study, we use the term “registered” to refer to PEPFAR applications or products that have been approved or tentatively approved by FDA. This should not be confused with the registration requirement of manufacturing facilities and listing of drug products that is required for all drugs distributed in the United States. Our analysis also included all changes made during the study time frame to the applications after initial registration (or postregistration changes), which often reflect product manufacturing or labeling updates or modifications.

To factor in the time FDA needed to initiate review or to take regulatory action, we assessed the regulatory status of the applications and postregistration changes in the study sample up to October 14, 2018. The analyses were conducted between October 2018 and February 2019.

This cross-sectional study used nonclinical data and did not involve human participants; therefore, we did not submit this study for human subjects ethics review. This study followed the Strengthening the Reporting of Observational Studies in Epidemiology (STROBE) reporting guideline for reporting on cross-sectional analyses.^11^

### Units of Analysis

This study used 2 primary units of analysis: applications and products. A third measure was used to assess postregistration changes, in which each change was considered a unique unit for our analysis.

An application, which can be an ANDA or an NDA, is a unique submission by a drug manufacturer seeking authorization from FDA to market specified product(s); each application may contain multiple products. For this study, a product was defined as an ART option that has 4 standard attributes, typically used by FDA to define a product: treatment options (either a single active ingredient or ≥2 active ingredients in fixed-dose combination or copackaged presentation), specific strength, specific dosage form (eg, solid, liquid, or pellet), and the manufacturer.

### Sampling Approach and Inclusion and Exclusion Criteria

We included applications that, at the time of analysis, were registered for PEPFAR use, had received a complete response letter (CRL), which contains FDA’s reasons for denying registration (applicants may resubmit an application and thus may receive more than 1 such letter), had been withdrawn after registration or issuance of a CRL, or were under review by FDA staff. We used registered applications that met the inclusion criteria to identify and study all unique modifications submitted for review as a postregistration change.

Applications were excluded that, at the time of analysis, were submitted to FDA but were withdrawn or canceled by the applicant before registration, were submitted to the FDA but had yet to start the review process, or were in refuse-to-file status (indicating applications that the FDA has declined to review because of incompleteness).^12,13^ Applications that were excluded could not have any postregistration changes, because they were never registered.

### Data Sources, Data Extraction, and Quality Check

Data on applications and postregistration changes were collected using 2 internal FDA databases: Document Archiving, Reporting & Regulatory Tracking System and the Center for Drug Evaluation and Research’s Informatics Platform. Data on WHO’s HIV treatment guidelines were collected from the 2016 update and from the July 2018 interim guidance.^9,14^ We also collected data on WHO first-line therapies that were preferred for use.

All data were quality-checked for accuracy and completeness through both manual and automated mechanisms. The manual checks were conducted by the primary data collectors and by FDA’s review experts responsible for managing PEPFAR applications. Inconsistencies in the data were rare (<5%) and, when found, were discussed and resolved.

### Statistical Analysis

We conducted 6 primary analyses: (1) an overview of the applications and products, including by year and key characteristics; (2) an analysis of the time to registration for registered products to determine the median time, in months, from submission of the application to FDA until the ART is available for procurement, including time spent on FDA review and all factors external to FDA review, such as time the applicant takes to respond to reviewer inquiries; (3) the number, types, and outcomes of requested changes made to PEPFAR applications after initial registration and the median time, in months, it takes for FDA to permit or approve those requests; (4) the number of CRLs, if applicable, as well as the reasons for the deficiencies, and the outcomes of those applications (the CRLs provide a proxy for the quality of the applications); (5) the association of the CRLs with time to registration for applications that were ultimately registered compared with applications that did not receive CRLs; and (6) an analysis of ARTs that can support the WHO’s current, 2018 preferred first-line HIV treatment recommendations.^9,14^

Data were analyzed using Excel 365 software version 2018 (Microsoft), Tableau Desktop software version 10.5 (Tableau), and, STATA IC statistical software version 13.1 (StataCorp). To account for nonparametric distribution of data for time to registration, we used median as our measure of central tendency and used the Mann-Whitney statistical test. Trend lines with 95% CIs and all statistical tests (2-sided) were conducted using STATA IC statistical software version 13.1 (StataCorp), with significance set at *P* = .05. All percentages were rounded to the nearest whole number for presentation.

## Results

### Overview

As of May 31, 2018, FDA had received 299 PEPFAR applications (encompassing 371 products). Of these, 260 applications (327 products) met the inclusion criteria and were eligible for analysis. Overall, 216 of the 260 applications were registered for use, of which 184 remain in active status and, thus, are available for use (Table 1). Fifty-six of 216 applications (26%) were for pediatric use, and the remainder were for adults.

**Table 1.** Overview of the President’s Emergency Plan for AIDS Relief Applications and Products Submitted to the US Food and Drug Administration From December 2004 to May 2018

Variable	No. (%)					
	Received and Reviewed		Registered at Any Time		Currently Available for Procurement	
	Applications (n = 260)	Products (n = 327)	Applications (n = 216)	Products (n = 272)	Applications (n = 184)	Products (n = 231)
Type of application						
Abbreviated New Drug Application^a^	161 (62)	211 (65)	128 (59)	169 (62)	116 (63)	155 (67)
New Drug Application^b^	99 (38)	116 (35)	88 (41)	103 (38)	68 (37)	76 (33)
Population						
Adult	200 (77)	247 (76)	160 (74)	196 (72)	138 (75)	169 (73)
Pediatric	60 (23)	80 (24)	56 (26)	76 (28)	46 (25)	62 (27)
Drug type						
Single drug	127 (49)	180 (55)	110 (51)	154 (57)	97 (53)	139 (60)
2-Drug fixed-dose combination	67 (26)	76 (23)	57 (26)	65 (24)	50 (27)	55 (24)
3-Drug fixed-dose combination	51 (20)	56 (17)	35 (16)	39 (14)	28 (15)	28 (12)
4-Drug fixed-dose combination	1 (<1)	1 (<1)	0	0	0	0
Copackaged drugs	14 (5)	14 (4)	14 (6)	14 (5)	9 (5)	9 (4)
Current regulatory status						
Registered	184 (71)	231 (71)	184 (85)	231 (85)	184 (100)	231 (100)
Under review	10 (4)	13 (4)	NA	NA	NA	NA
Complete response letter	12 (5)	14 (4)	1 (<1)	1 (<1)	NA	NA
Withdrawn	54 (21)	69 (21)	31 (14)	40 (15)	NA	NA
Applicant country						
China	4 (2)	4 (1)	4 (2)	4 (1)	3 (2)	3 (1)
India	244 (94)	305 (93)	201 (93)	251 (92)	174 (95)	217 (94)
South Africa	8 (3)	11 (3)	8 (4)	11 (4)	5 (3)	6 (3)
United States	4 (2)	7 (2)	3 (1)	6 (2)	2 (1)	5 (2)

Abbreviation: NA, not applicable.

^a^ Abbreviated New Drug Applications are filed for duplicates of existing antiretroviral therapies.

^b^ New Drug Applications are filed for new variations of existing antiretroviral therapies.

At the product level, of the 327 products reviewed by FDA, 83% (272) were registered for use by PEPFAR (Table 1), whereas the remainder were withdrawn before registration, were found to be deficient, or were under review at the time of analysis. Of these 272 products, 231 (85%), including 62 pediatric-specific products and 83 fixed-dose combination products, remain in registered status, indicating availability for procurement by PEPFAR and other entities (Table 1).

Among the 231 products available at the time of analysis, 155 (67%) were registered under ANDAs (duplicates of an ART previously approved for use in the United States), whereas the remaining 76 (33%) were reviewed under NDAs (new combinations, strengths, or formulations of existing products) (Table 1). Most (169 [73%]) were intended for adults; the remainder were pediatric-specific strengths or formulations.

**Figure 1.**
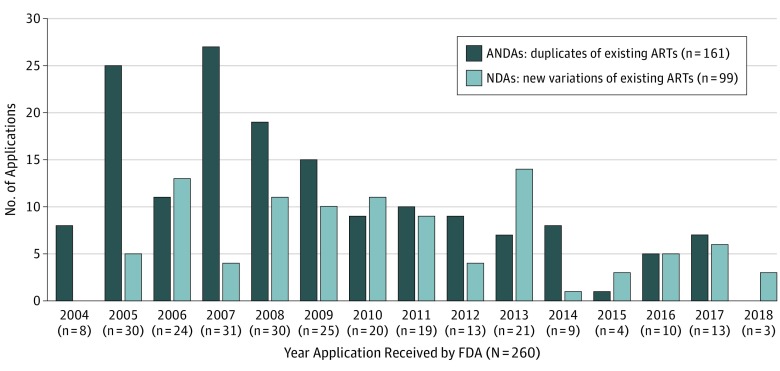
US President’s Emergency Plan for AIDS Relief Applications Submitted to the US Food and Drug Administration (FDA) Since 2004, by Application Type The data for 2018 are partial, up to May 2018. Applications are shown regardless of current regulatory status. ANDA indicates Abbreviated New Drug Application; ART, antiretroviral therapy; and NDA, New Drug Application.

Despite a noticeable increase in number of applications submitted in 2013, overall, the number of PEPFAR applications has decreased since its peak between 2005 and 2008 (Figure 1). The trend in submission of applications for new variations (NDAs) vs duplicates of existing drugs (ANDAs) has fluctuated over time, with no clear pattern. The 2013 increase was associated with NDA submissions. Overall, single-molecule drugs were the most common (55%) form of ARTs submitted for review (Table 1); however, since 2011, 2-drug, 3-drug, and 4-drug fixed-dose combinations collectively make up a larger portion of submissions than single-molecule drugs (61% vs 33%).

### Analysis of Time to Registration

In the analysis of time to registration, we included only the 216 of 260 applications that had achieved registration at any time, regardless of their current application status. The remaining applications were not eligible for the analysis of time to registration because they had not received registration at the time of analysis.

**Figure 2.**
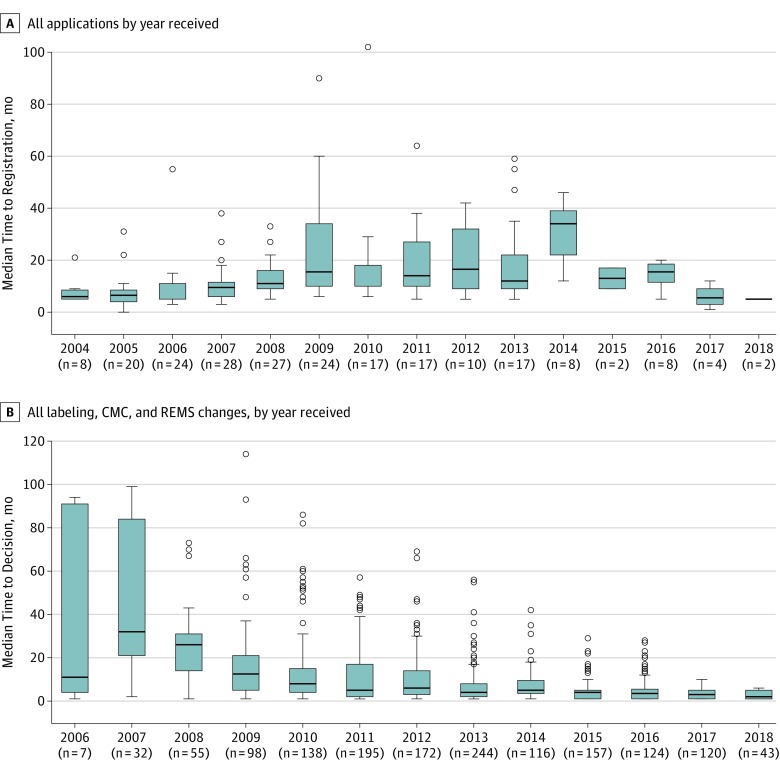
Time to Registration and Time to Decision for Applications The data for 2018 are partial, up to May 2018. A, Box plots show the median time to registration (overall median [interquartile range], 10.0 [7.0-17.5] months), which may include multiple review cycles and is inclusive of all time from the day the US Food and Drug Administration (FDA) receives an application for review to the day it is made available for use by the US President’s Emergency Plan for AIDS Relief. As such, the time includes FDA's review time as well as any time spent by the application holder in providing or responding to FDA questions related to the review. For example, denial of an application will inevitably increase time to registration because the application will require multiple review cycles; other factors, such as submission of new information by the applicant toward the end of review process, may extend the review time to give the FDA more time to consider the new information. B, Box plots show the time to decision by FDA on postregistration changes over time (overall median [interquartile range], 5.0 [2.0-12.0] months). The decision in this analysis is an affirmative decision to allow the requested change. Submissions for postregistration changes started in 2006 and since then the time to decision has consistently decreased from a median of 10.0 to 30.0 months to 5.0 months. Lines within boxes denote medians; bottom and top borders of boxes denote the 25th and 75th percentiles, respectively; vertical lines denote interquartile ranges; and circles denote outliers. CMC indicates Chemistry, Manufacturing, and Controls; REMS, Risk Evaluation and Mitigation Strategy.

For all 216 applications, the median (interquartile range [IQR]) time to registration was 10.0 (7.0-17.5) months, with fluctuations observed over the years and longer times for applications received between 2009 and 2014 (Figure 2A). The time to registration for single-drug applications was slightly longer (median [IQR], 11.0 [7.0-19.0] months) compared with applications for fixed-dose combination drugs (median [IQR], 10.0 [6.5-18.0] months). A similar pattern was seen in applications for duplicates of existing ARTs (ANDAs) (median [IQR], 13.5 [8.0-26.5] months) vs applications for new variations (NDAs) (median [IQR], 10.0 [5.5-10.0] months).

### Changes to Applications After Registration and Time to Decision

A total of 1810 requests for postregistration changes were submitted to FDA for the 216 applications that achieved registration; 79% (1430) of these changes were associated with drug manufacturing processes, 20% (370) were for drug labeling, and the remaining 10 (<1%) were for postmarket safety evaluation plans (called risk-evaluation and mitigation strategy). Eighty-three percent (1501) of the requested changes were permitted, 11% (193) were withdrawn by the applicant, and the remaining 5% (88) were still under review or had been denied (28 [2%]). Although trends varied, a median (IQR) of 136 (86-192) changes were submitted annually over the study period.

Among the permitted changes, it took FDA a median (IQR) of 5 (2-12) months to review a postregistration change (Figure 2B). Reviews for labeling changes were typically faster, at a median (IQR) of 3 (1-12) months compared with reviews for manufacturing changes, with a median (IQR) of 5 (3-12) months. Review time was shortest for the 10 risk-evaluation and mitigation strategy changes with a median (IQR) of 1 (1-4) month.

### Reasons for Denying Registration and Outcomes

Study applications received a total of 172 CRLs (deficiency letters). Ninety-five of 260 applications (37%, both ANDAs and NDAs) received at least 1 CRL, but some applications had as many as 6 CRLs (Table 2). Single-molecule drugs received the most CRLs (80 [47%]), followed by 3-molecule drugs (46 [27%]) and 2-molecule drugs (43 [25%]).

**Table 2.** Applications With CRLs and Their Reasons and Outcomes, Stratified by Each Review Cycle^a^

Variable	Applications, No. (%)^b^						
	1 CRL	2 CRLs	3 CRLs	4 CRLs	5 CRLs	6 CRLs	Total CRLs Issued
Drug type							
Single drug	45 (47)	21 (50)	9 (45)	3 (38)	2 (33)	NA	80 (47)
2-Drug fixed-dose combination	23 (24)	10 (24)	6 (30)	2 (25)	1 (17)	1 (100)	43 (25)
3-Drug fixed-dose combination	24 (25)	11 (26)	5 (25)	3 (38)	3 (50)	NA	46 (27)
4-Drug fixed-dose combination	1 (1)	NA	NA	NA	NA	NA	1 (1)
Copackaged drugs	2 (2)	NA	NA	NA	NA	NA	2 (1)
Total	95 (100)^c^	42 (44)^c^	20 (21)^c^	8 (8)^c^	6 (6)^c^	1 (2)^c^	172 (100)
Reasons for issuing a CRL							
Manufacturing and chemistry	67 (44)	23 (37)	15 (60)	5 (45)	4 (40)	1 (33)	115 (44)
Labeling	36 (24)	18 (29)	4 (16)	1 (9)	2 (20)	1 (33)	62 (23)
Facility inspection	24 (16)	18 (29)	4 (16)	5 (45)	3 (30)	NA	54 (20)
Bioequivalence	18 (12)	3 (5)	2 (8)	NA	NA	NA	23 (9)
Biopharmaceutics	6 (4)	NA	NA	NA	NA	NA	6 (2)
Missing facility	1 (1)	NA	NA	NA	NA	NA	1 (<1)
Packaging	1 (1)	NA	NA	NA	NA	NA	1 (<1)
Risk evaluation and mitigation strategy^d^	NA	NA	NA	NA	1 (10)	1 (33)	2 (1)
Total	153 (59)^c^	62 (24)^c^	25 (10)^c^	11 (4)^c^	10 (4)^c^	3 (3)^c^	264 (100)
Outcomes of applications							
Registered	34 (36)	11 (26)	5 (25)	1 (13)	1 (17)	NA	52 (55)^e^
Withdrawn	12 (13)	9 (21)	2 (10)	NA	NA	NA	23 (24)
Remain in CRL status or no resubmission	1 (1)	2 (5)	3 (15)	1 (13)	3 (50)	1 (100)	11 (12)
Resubmission or under review	6 (6)	1 (2)	2 (10)	NA	NA	NA	9 (9)
Received another CRL	42 (44)	19 (45)	8 (40)	6 (75)	2 (33)	NA	NA^f^
Total	95 (100)^c^	42 (44)^c^	20 (21)^c^	8 (8)^c^	6 (6)^c^	1 (2)^c^	95 (100)

Abbreviations: CRL, complete response letter; NA, not applicable.

^a^ A CRL is a letter issued by the US Food and Drug Administration listing deficiencies in an application that prevent its registration.

^b^ All percentages are column based (top to bottom), unless otherwise stated.

^c^ Row percentages (left to right).

^d^ A risk evaluation and mitigation strategy is a plan for postmarket drug safety surveillance.

^e^ Included 1 application that was registered after a CRL, but the registration was rescinded and remains in CRL status.

^f^ Totals exclude the “Received another CRL” category. This category contains double-counted applications; thus, it was omitted to prevent incorrect counts.

Among all CRLs in our sample, a total of 264 reasons for denying registration were identified (Table 2). The most common reason was deficiencies in manufacturing processes (155 [44%]), followed by product labeling (62 [23%]) and failed facility inspections (54 [20%]). The product labeling concerns were observed only in ANDAs (applications for duplicate ARTs).

Ultimately, 52 of 95 applications (55%) received registration after addressing their deficiencies; 34 were registered after receiving only 1 CRL. However, 23 of 95 applications (24%) were withdrawn by the applicant, including 12 after the first CRL. Nine applications were under review at the time of analysis and 11 were with the applicant (in CRL status) (Table 2).

### Association of Denials With Time to Registration

**Figure 3.**
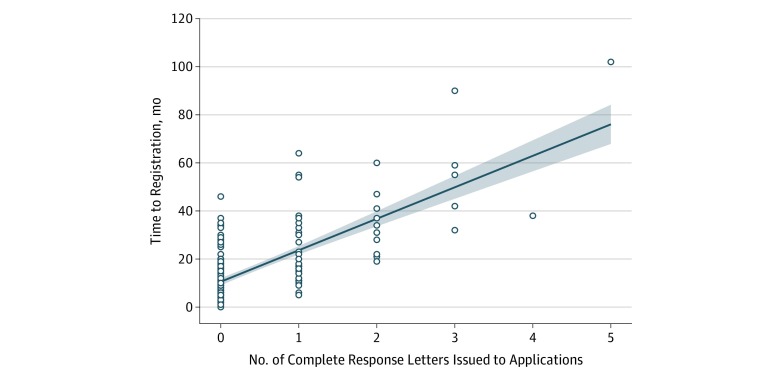
Complete Response Letters Issued to Applications Scatterplot with applications (circles), trend line, and 95% CIs (shaded areas) shows that as the number of complete response letters issued to applications increases, so does the time to registration.

We analyzed 51 of the 52 applications that received registration after addressing their deficiencies and compared their time to registration with the 164 applications that did not receive any CRLs (Figure 3). For consistency, we excluded 1 application because of its unique regulatory history; the product was registered after receiving CRLs, but the registration was later rescinded and had not been reregistered at the time of analysis.

As expected, applications with CRLs typically took longer to achieve registration than those without. Applications that did not receive any CRLs took a median (IQR) of 9.0 (5.5-12.0) months to get to market, whereas those with at least 1 CRL required a median (IQR) of 22.0 (14.0-38.0) months. A Mann-Whitney test of the difference in median time to registration between applications that received no CRLs compared with those that received at least 1 such letter was found to be significant (9.0 months vs 22.0 months; *P* < .001).

### ARTs Supporting Preferred First-Line WHO Treatment Recommendations

Sixty-one percent (140 of 231) of the currently available ARTs registered by the FDA can support WHO’s first-line recommended therapies; of these, 44 products are pediatric-specific strengths and formulations. Seven of the 140 are dolutegravir options, a new preferred first-line ART. Nineteen first-line drugs, including 4 dolutegravir-based options, were either under review at FDA or were in CRL (deficient) status at the time of analysis.

## Discussion

This analysis of FDA’s PEPFAR program found that 272 ARTs have been registered for use in resource-constrained settings. Two hundred thirty-one of these were available for procurement at the time of analysis, including 62 pediatric-specific products and 83 fixed-dose combination products.

We observed a decrease in the number of PEPFAR applications submitted annually since the beginning of the program; this can likely be explained by 4 factors. First, market saturation and increased competition among clinically needed drugs might have reduced market incentives for new applicants; at the time of analysis, there were 140 products available that supported WHO’s first-line treatment recommendations. Second, there have been consistent efforts by the HIV community to develop and use fixed-dose combinations for treatment because of their ease of use, improved patient adherence, and cost-effective procurement, transportation, and storage.^15,16^ Our data show that, although FDA still receives single-drug applications, in recent years, the fixed-dose combinations have constituted a higher fraction of submissions, potentially leading to fewer overall applications while still meeting clinical needs. Third, the US Department of State’s active role in working with manufacturers to procure only clinically necessary drugs could reduce a manufacturer’s incentive to undergo FDA review for products not meeting procurement criteria.^17^ An in-depth assessment of the outcomes of PEPFAR procurement criteria on submissions was beyond the scope of this study. Fourth, FDA did not charge user fees for ANDAs before October 2012; under a new law, FDA is now required to charge certain fees for all ANDAs.^4,18^ It is possible that some manufacturers have chosen to be more strategic about their PEPFAR submissions as a result of the new fees. However, a similar decrease was also observed for NDA applications, and FDA typically waives its fees for these submissions.^4^ Any of these factors could explain our findings.

Furthermore, our analysis found that FDA reviewed and registered most PEPFAR NDAs and ANDAs within 10.0 months and 13.5 months of receiving an application, respectively; this includes time spent outside the agency. These findings are broadly consistent with the latest performance of all FDA-reviewed applications. In 2017, the median time to approve NDAs was 7.0 to 10.0 months, depending on the type of NDA, whereas the median time for ANDA approval was 13.7 months.^19,20^

Our study also found that FDA uses a robust quality check to ensure safety and efficacy of the products it registers. Thirty-seven percent of all PEPFAR applications received at least 1 CRL, primarily because of deficiencies in manufacturing processes or other quality-related issues at the manufacturing facility. Furthermore, 23% of CRLs were issued for labeling deficiencies (all ANDAs), indicating a potential for inadequate instructions or packaging that may interfere with correct use of the product. To catalog the quality issues encountered with PEPFAR applications, we looked only at the issues identified in the CRLs; we did not evaluate the numerous other issues that are often identified and resolved during the review process without issuance of a CRL. In addition, our analysis indicates that receipt of a CRL can substantially delay registration or lead to withdrawal of applications.

To our knowledge, this is the first time FDA CRLs of noninnovator products have been studied. Previous studies have primarily focused on innovator drugs, particularly new molecular entities (drugs never before registered in the United States).^21,22^ Because of their focus on innovative drugs, previous literature has primarily identified safety and efficacy as the main reasons why FDA denies marketing approval. This is expected because new drugs require clinical trials, in which safety and efficacy are evaluated. Applications to FDA’s PEPFAR pathway, however, rely on existing drugs that are already approved for use in the United States; therefore, it is unlikely that new clinical safety and efficacy issues would arise during review.^4^

The FDA’s PEPFAR program remains on the cutting edge of treatment needs and continues to support first-line treatment options for HIV. For example, in July 2018, WHO issued an amendment to its 2016 HIV guidelines to include a new ART called dolutegravir,^9^ which in combination with other ARTs, has fewer adverse effects, may help achieve viral control faster, and is cheaper. Because dolutegravir could be used for patients who are currently receiving other ARTs (including second-line therapies), it could simplify supply chains by reducing the number of drugs for which procurement is needed.^23^ Although the use of dolutegravir by women at the time of conception has led to some concern about the potential for serious fetal adverse effects, the drug is still considered important for controlling the HIV epidemic, and PEPFAR and the global HIV community have made dolutegravir availability central to their strategy to combat HIV.^23,24,25,26,27^ To help achieve this goal, and with continued assessments of drug safety, FDA has already registered 7 dolutegravir-based products, and more are under review. Since the beginning of the program, FDA has encouraged the development of new, effective ART combinations by providing guidance to manufacturers, and will continue to do so.^5^

The FDA, HIV community, and drug companies can improve collaboration in some areas to enhance access to live-saving therapies. One such area is development of, and access to, better therapies for children.^16^ In 2018, there were 160 000 new pediatric infections of HIV and 1.7 million children living with HIV globally, of whom only 54% were receiving therapy.^1^ Many of the currently available HIV medicines for children are either tablets or bitter-tasting syrups, which make them difficult to give to children; furthermore, syrups that require refrigeration pose procurement and storage challenges in resource-limited settings.^28,29^ Although FDA has registered many pediatric-specific ARTs, such as 2-drug combination pellets that can be sprinkled on food for ease of administration, more are needed, in particular 4-drug combinations to further simplify pediatric therapy, as well as tablets for oral suspension, ideally with masked taste.^28,30^ The FDA recently issued a guidance to help manufacturers develop drugs to treat pediatric HIV and the agency stands ready to work with drug companies and the HIV community to advance the availability of pediatric drugs for global use.^6^

Because most applications (55%) with a CRL in our sample ultimately achieved registration, it may be possible for applicants to avoid some common, preventable application quality deficiencies. Manufacturers may consider asking for agency input on certain key application issues before submission to improve the application quality and potentially avoid a CRL. Written responses to questions, teleconferences, or meetings are sometimes available to applicants needing feedback before submitting applications.^31^

Continued efforts to reduce duplication of drug reviews between FDA and the WHO drug review mechanism (prequalification of medicines program), as identified by previous research,^8^ are needed. As an initial step, to reduce duplication and increase timely access to ARTs in countries of greatest need, FDA and WHO have initiated a joint pilot program in which FDA will share its completed drug reviews, to determine whether this will expedite reviews of the same products by WHO’s prequalification of medicines program.^32^ The resulting review dossiers from WHO’s prequalification of medicines program can, in turn, be shared with regulatory agencies in resource-limited countries to speed up their own review processes, helping patients access HIV treatment faster.

### Limitations

This study had several limitations. First, we did not analyze whether the FDA-registered HIV drugs studied in this analysis are clinically needed; however, as a proxy for clinical importance, we conducted an analysis to determine what fraction of the drugs supported WHO’s first-line HIV treatment guidelines. Second, we did not determine whether the FDA-registered products are being actively manufactured or procured. We only considered whether the drugs were registered and in good regulatory standing with the FDA to allow for manufacturing or procurement. Third, we only reviewed high-level reasons for why FDA rejected some applications. For example, we did not assess the specific type of manufacturing or facility problems that resulted in the rejections. An in-depth evaluation of all technical reasons for rejections was beyond the scope of this study; however, an evaluation of detailed reasons may be helpful to applicants to improve the application quality. In addition, because this study was a focused evaluation of FDA’s PEPFAR program, the findings of this study cannot be generalized to other drugs reviewed by the agency.

## Conclusions

Since its inception, FDA’s PEPFAR program has made 272 ARTs available for global use, 140 of which currently support WHO’s first-line HIV treatment preferences. The FDA continues to support the global HIV community’s efforts by quickly reviewing and registering new, safer, and more effective ARTs. The FDA and pharmaceutical companies should explore steps to improve the quality of applications submitted to prevent avoidable deficiencies in manufacturing processes and labeling. International efforts need to continue to develop better, easier to use pediatric-specific HIV therapies.
